# Growth hormone response to submaximal doses of ghrelin remains unchanged during the follicular phase of the cycle

**DOI:** 10.1186/1477-7827-11-36

**Published:** 2013-05-10

**Authors:** Christina I Messini, Konstantinos Dafopoulos, Maria Malandri, Panagiotis Georgoulias, George Anifandis, Ioannis E Messinis

**Affiliations:** 1 Department of Obstetrics and Gynaecology, School of Health Sciences, Faculty of Medicine, University of Thessalia, Larissa, Greece; 2 Department of Nuclear Medicine, School of Health Sciences, Faculty of Medicine, University of Thessalia, Larissa, Greece

**Keywords:** Ghrelin, Growth hormone, Estradiol, Menstrual cycle

## Abstract

**Background:**

Previous data have shown that ghrelin-induced growth hormone (GH) secretion is augmented in women by exogenous but not by endogenous estrogens. The purpose of this study was to examine the response of GH to low-dose scheme of ghrelin administration in relation to physiological changes in estradiol levels during the normal menstrual cycle.

**Methods:**

Ten normally cycling women were studied in two menstrual cycles. Two consecutive dosages of ghrelin (0.15 μg/kg and 0.30 μg/kg) were injected intravenously at 0 and 90 min in the early and late follicular phases of one cycle. Saline was injected in the preceding cycle. Blood samples were taken at −15, 0, 30, 60, 90, 120, 150 and 180 min. The GH response was assessed.

**Results:**

Serum estradiol concentrations were significantly higher in the late than in the early follicular phase. After ghrelin, but not after saline administration, plasma ghrelin and serum GH levels increased significantly in both phases, peaking at 30 min and 120 min. The peak value at 120 min was significantly higher than at 30 min (P<0.001). There were no significant differences in ghrelin and GH levels between the two phases at all time points.

**Conclusions:**

The present results show no difference in GH response to two consecutive submaximal doses of ghrelin between the early and the late follicular phase of the cycle. It is suggested that estradiol is not possibly involved in the physiological process that regulates ghrelin-induced GH secretion in women during the normal menstrual cycle.

## Background

Ghrelin is a 28-amino acid protein, which is produced in the mucosa of the stomach but it is also expressed in other tissues. This substance is considered the endogenous ligand for the growth hormone secretagogue receptor (GHS-R) [1]. Various experiments in animals and humans have shown that ghrelin stimulates the secretion of GH together with other pituitary hormones, such as prolactin and adrenocorticotrophic hormone [2]. A role for ghrelin in the regulation of reproductive function has been suggested both in animals and in humans [3-5]. A possible site of action is the ovaries [6,7], affecting steroidogenesis in humans [8], while an inhibitory effect on LH and FSH secretion has been shown in animals, men and women [9-13].

Previous studies have shown that acylated and unacylated ghrelin and GH levels do not change significantly during the normal menstrual cycle [14,15]. It has been also demonstrated that the stimulating action of ghrelin on GH secretion is affected by ovarian steroids. This is based on experiments performed in postmenopausal women treated with the exogenous administration of estradiol or the combination of estradiol plus dydrogesterone [16-18]. In the two studies with estradiol alone, an enhancing effect of this steroid on ghrelin-induced GH secretion was observed with the use of either a single submaximal dose of ghrelin [16] or multiple dosages in the context of a step-wise, every 90 min, increase [17]. A more recent study, however, in normally cycling women has not shown any difference in the GH response to a single relatively high dose of ghrelin among three different phases of the cycle despite marked changes in ovarian steroids concentrations including estradiol [15]. Whether, under these conditions, low-dose schemes of ghrelin stimulation, as compared to a single high dose, could provide different GH responses, has not been investigated.

The present study was undertaken to investigate the hypothesis that the effect of two consecutive submaximal doses of ghrelin on GH secretion in the early and the late follicular phase of the menstrual cycle is not affected by changes in the physiological concentrations of estradiol in normal women.

## Methods

### Subjects

Ten normally cycling women (aged 22.8±3.7 years, BMI 21.0±3.4 kg/m^2^, mean±SD) were included. The length of the menstrual cycle was 27–28 days. The women volunteered for the study and gave written informed consent. Approval for the study was obtained from the local Ethical Committee. Ovulation was confirmed before the onset of the study by serum progesterone measurement and ultrasound scans of the ovaries. The women were not taking any hormonal or any other medical treatment for the last 6 months before entering the study. They had normal thyroid and adrenal function, while serum prolactin levels were within the normal range. All women were investigated during two menstrual cycles. In each cycle the same experimental procedure was performed twice, i.e. in the early follicular phase (cycle day 2) and in the late follicular phase, when the size of the dominant follicle, as assessed by ultrasound reached the diameter of 16–17 mm. In both cycles, after overnight fasting, the women were admitted to the clinical research unit around 0930 h. An indwelling cannula was inserted in the forearm vein and the women were left to relax for about 15 min. Then, a blood sample was taken (time −15 min) and a second one 15 min later (time 0 min). Immediately after the second blood sample was obtained, in the second cycle (study cycle) a single dose of 0.15 μg/kg ghrelin was given to the women as an acute i.v. injection. Further blood samples, in relation to time 0, were taken at 30, 60, 90, 120, 150 and 180 min. A second dose of 0.30 μg/kg ghrelin was injected immediately after the 90 min blood sample. The same experimental procedure was performed twice in the first cycle preceding the study cycle with the difference that instead of ghrelin, 2 ml of normal saline was injected i.v. at 0 and 90 min (control cycle). All blood samples were centrifuged at 1000 g for 15 min and serum and plasma were stored at −20°C until assayed. In all blood samples, GH was measured. Ghrelin was measured in the samples from −15 to 120 min inclusive, while estradiol and progesterone were measured only in the 0 min sample. No side effects were seen in any of the women following the administration of ghrelin.

### Hormone assays

Measurement of total ghrelin in plasma was performed using a radioimmunoassay (KIPMR90, DIASource ImmunoAssays S.A., Louvain-la-Neuve, Belgium) and the results are expressed as pg/ml. Measurement of GH in serum was performed using an immunoradiometric assay (hGH-IRMA, IMMUNOTECH S.A.S., Marseille Cedex, France) and the results are expressed as ng/ml. Estradiol was measured in serum using a radioimmunoassay (RIA-ESTRADIOL, IMMUNOTECH S.A.S., Marseille Cedex, France). The results are expressed as pg/ml. Measurement of progesterone in serum was performed with the use of a radioimmunoassay (PROG-RIA-CT, DIASourse ImmunoAssays S.A., Louvain-la-Neuve, Belgium). The results are expressed as ng/ml. The lower limits of detection for ghrelin, GH, estradiol and progesterone were 40 pg/ml, 0.03 ng/ml, 6.0 pg/ml and 0.05 ng/ml respectively. Inter- and intra-assay coefficients of variation were 7.1 and 4.2%, 8.7 and 0.9%, 8.5 and 7.5% and 6.8 and 4.2% respectively.

### Statistical analysis

Hormone values were normally distributed (one sample Kolmogorov-Smirnov test) and statistical analysis was performed by repeated measures one-way analysis of variance (ANOVA) followed by Bonferroni post hoc testing and paired t-test. An *a* level of 0.05 was used to determine statistical significance. All values are expressed as mean±SD. The statistical software package used was NCSS 2001 (Number Cruncher Statistical Systems, Kaysville, UT, USA).

## Results

Serum estradiol concentrations on day 2 were similar in the two cycles (control cycle: 30.5±5.0 pg/ml, study cycle: 26.1±4.6 pg/ml). Follicle size (mean diameter of two measurements) and serum estradiol values (time 0 min) on the day of the experiment in the late follicular phase were similar in the two cycles (16.4±0.2 and 16.5±0.2 mm and 133.1±22.6 and 126.5±23.4 pg/ml respectively). Estradiol values in the late follicular phase were significantly higher than in the early follicular phase in both cycles (P<0.001). Serum progesterone values were low in both phases of the cycle with no difference between the two cycles (mean value <1 ng/ml).

**Figure 1:**
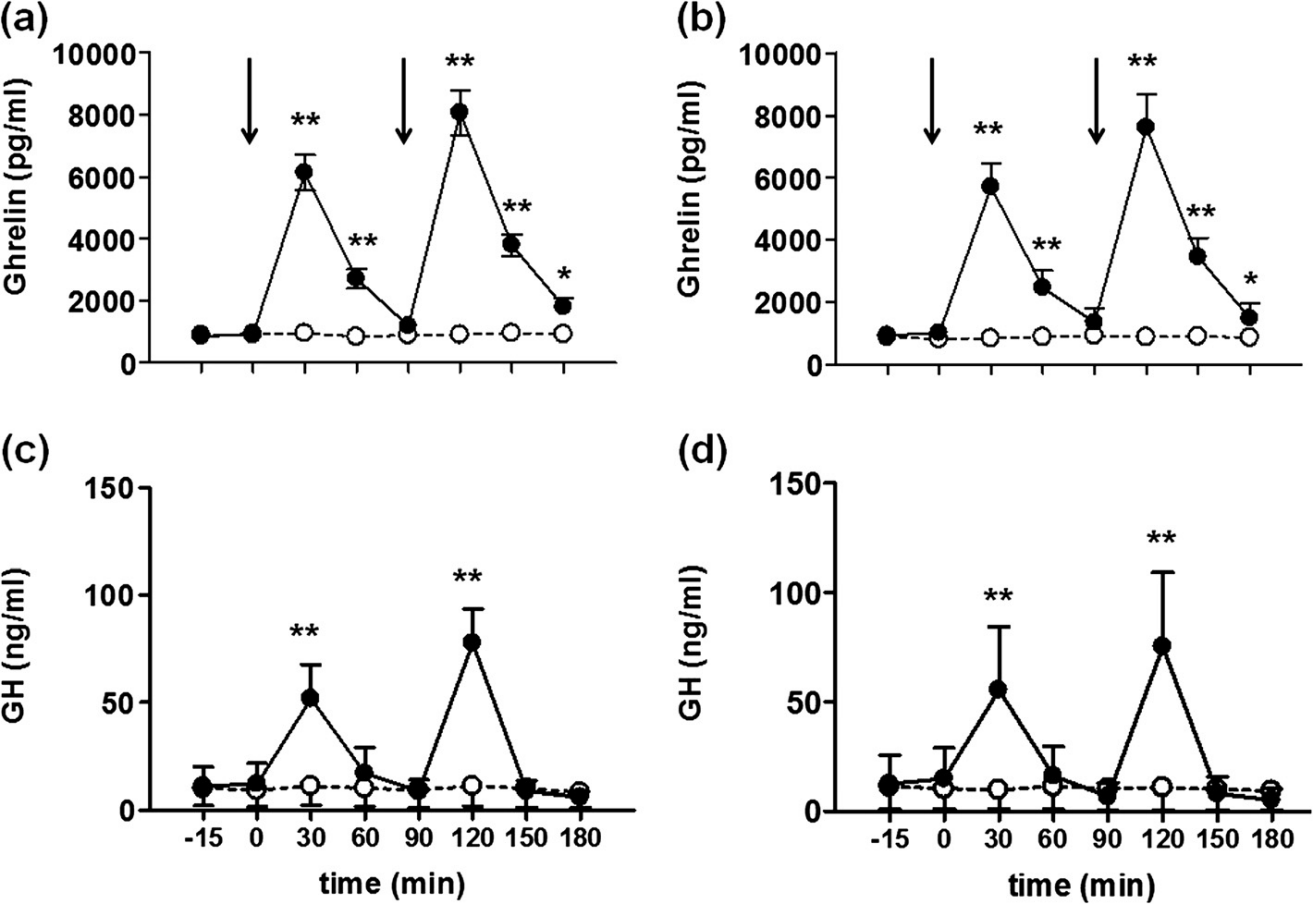
**Plasma ghrelin and serum GH values in (a and c) early (cycle day 2) and (b and d) late follicular phase of the cycle (follicle size 16–17 mm) in 10 normally cycling women before (time −15 and 0 min) and after the i.v. injection (arrows) of (●) ghrelin (0.15 μg/kg at 0 min and 0.30 μg/kg at 90 min) or (o) saline (2 ml at 0 and 90 min).** **P<0.001, *P<0.05 (difference from saline experiments).

**Figure 2:**
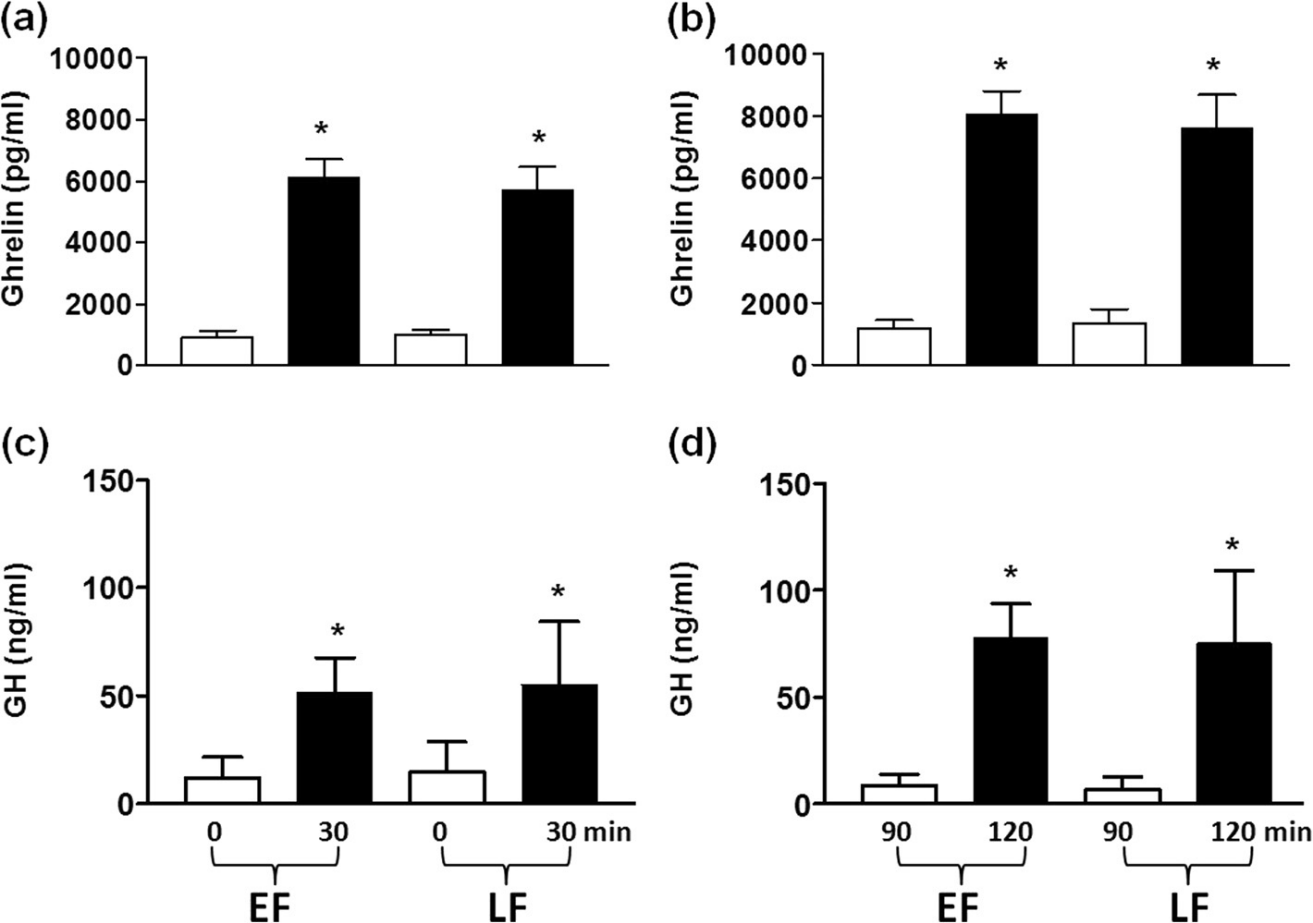
**Plasma ghrelin and serum GH values in early (EF) and late follicular phase of the cycle (LF) in 10 normally cycling women (□) before (time 0 and 90 min) and (■) after (time 30 and 120 min) the i.v. injection of ghrelin at the dose of (a and c) 0.15 μg/kg and (b and d) 0.30 μg/kg.** **P<0.001 (difference between 30 and 0 min and between 120 and 90 min).

Figure 1 shows plasma ghrelin and serum GH levels during the two experiments. Plasma ghrelin concentrations were similar in the two cycles both in the early (Figure 1a) and the late follicular phase (Figure 1b) before the onset of the experiment (time 0 min). Ghrelin values increased significantly after each ghrelin injection. Peak values were seen at 30 min (early follicular: 6049±181, late follicular: 5703±362 pg/ml) and 120 min (early follicular: 8010±228, late follicular: 7613±370 pg/ml), but the peak at 120 min was significantly higher than at 30 min in both phases (P<0.001). Plasma ghrelin values were significantly lower at 90 min than at 30 min and at 150 and 180 min than at 120 min in both phases (P<0.001) (Figure 1). The peak values of ghrelin at 30 and 120 min in the early follicular phase were similar to those in the late follicular phase respectively (Figure 2a and b). In the control cycle, plasma ghrelin values did not change significantly following the administration of saline but remained stable throughout the experimental procedure in both phases with no difference between them at all time points (Figure 1). Plasma ghrelin concentrations were significantly higher in the study cycle than in the control cycle at 30, 60, 120 and 150 min (P<0.001) as well as at 180 min (P<0.05) in both phases (Figure 1a and b).

Serum GH values were similar before the onset of the experiment (time 0 min) in the two phases of the cycle both in the control and the study cycle (Figure 1). Following the first injection of ghrelin, serum GH levels increased significantly at 30 min both in the early (51.0±5.1 ng/ml, P<0.001, Figure 1c) and the late follicular phase (55.3±4.7 ng/ml, P<0.001, Figure 1d). Then, GH values returned to the baseline at 60 and 90 min (P<0.001), but increased again significantly at 120 min following the second injection of ghrelin in the early (76.0±5.0 ng/ml, P<0.001, Figure 1c) and the late follicular phase (75.2±11.3 ng/ml, P<0.001, Figure 1d). The peak value at 120 min was significantly higher than the peak value at 30 min in both phases of the cycle (P<0.001). A significant decrease in GH levels was seen at 150 and 180 min as compared to the 120 min levels in both phases of the cycle (P<0.001, Figure 1c and d). The peak values of GH at 30 and 120 min in the early follicular phase were similar to those in the late follicular phase respectively (Figure 2). In the saline experiments, serum GH values remained constant with no significant difference between the two phases at all time points. Serum GH values at 30 and 120 min were significantly higher in the study cycle than in the control cycle in both phases (P<0.001) (Figure 1c and d). The pattern of changes in serum GH values was in both phases of the study cycle almost identical to those of plasma ghrelin values.

## Discussion

The present study confirms that the exogenous administration of ghrelin stimulates in women the secretion of GH leading to a significant increase in serum concentrations of this hormone [2,15]. In this study, however, we used two submaximal but still pharmacological doses of ghrelin and found that the response of GH was dose-dependent. Previous studies in postmenopausal women have shown that in an artificial ‘simulated follicular phase’, created after the exogenous administration of estrogens, the stimulating effect of a single or multiple doses of ghrelin on GH secretion was enhanced as estradiol concentrations increased [16,17]. This, however, was not confirmed in a more recent study in which the stimulating action of a single dose of ghrelin (1 μg/kg) on GH secretion was not affected by changes in the endogenous hormonal profile, such as the increasing concentrations of estradiol during the follicular phase of the cycle [15]. It was considered then, that multiple submaximal dosages of ghrelin might be more physiological than a single high dose. Nevertheless, this does not seem to be the case, since in the present study using submaximal dosages of ghrelin, we did not find any significant difference in GH response between the two stages of the cycle. In addition, when ghrelin was given in a previous study as a bolus at the dose of 1 μg/kg to a group of normal postmenopausal women treated for 60 days with the combination of 2 mg estradiol and 10 mg dydrogesterone, the GH response to that dose was also enhanced by these steroids [18]. It is suggested, therefore, that it is not the amount of ghrelin that accounts for the different results between the present and the previous studies.

It should be emphasized that the enhanced response of GH secretion to ghrelin in the previous studies was seen after the administration of exogenous estrogens, while in this study we took into account only endogenous estrogens. In addition to that, in the previous studies [16,17], the role of endogenous estrogens was not investigated, while the women who were included were postmenopausal. However, the model of postmenopausal women may not be the most appropriate, since due to the long-term oestrogen deprivation, the integrity of the hypothalamic-pituitary system may have been compromised. Previous data have shown a reduction in the circulating concentrations of GH with ageing [19], while the response of GH to ghrelin was attenuated in the elderly as compared to young male or female subjects [20]. It is possible, therefore, that the enhanced response of GH to ghrelin in postmenopausal women following the administration of oestrogens is explained on the basis of a restoration of the impaired pituitary function by these steroids, which rendered the pituitary cells more sensitive to the different secretagogues. In addition to these, previous data have shown that postmenopausal women demonstrate a reduced GH response to various secretagogues as compared to premenopausal women [21]. It has been shown previously that in experiments involving the administration of estradiol in combination with exogenous ghrelin to postmenopausal women, it was possible to predict nighttime production rates of acylated ghrelin, in the context of increased synthesis and/or acylation of ghrelin peptide by estradiol [22]. Whether endogenous estrogens could have a similar effect on ghrelin synthesis has not been investigated.

Regarding the use of GH secretagogues other than ghrelin, inconsistent results have been obtained. In the majority of the studies, the GH response to such substances was enhanced by exogenous estradiol even if the dose utilized was rather high. For instance, the response to a single dose of 3 μg/kg of GHRP-2 was enhanced by estradiol treatment in postmenopausal women [23]. In contrast, an enhanced response of GH was not seen when hexarelin was used as a stimulus at the dose of 2 μg/kg and the women were treated with transdermal estradiol for three months [24]. Nevertheless, in none of these studies the role of endogenous estrogens was investigated.

It is likely that the present experiments, performed in the presence of naturally increasing levels of estradiol during the follicular phase of the cycle, provide a more physiological way to investigate the role of estrogens on the endocrine actions of ghrelin rather than during a ‘simulated’ mode of estradiol increase. Certainly, this is a pharmacological study and not necessarily applying to the physiology. The investigation of the pulsatile secretion of acylated ghrelin in conjunction with GH secretion might be more appropriate, but this could be the aim of a new study with a different design. Although it might be that GH secretion in response to ghrelin is controlled differentially by endogenous and exogenous ovarian steroids [15], understanding the role of ghrelin on GH secretion during the normal menstrual cycle will certainly require a sophisticated rather than a simplified approach, as it is the use of various pharmacological tools.

## Conclusions

The present results show for the first time that the response of GH to two consecutive submaximal doses of ghrelin was similar in the early and the late follicular phase of the cycle. Since great hormonal differences occur between these two stages, it is suggested that estradiol is not possibly involved in the physiological process that regulates ghrelin-induced GH secretion in women during the normal menstrual cycle.

## Competing interests

The authors declare that they have no competing interests.

## Authors’ contribution

CIM carried out the experiments and wrote the first draft. KD participated in the experimental design and statistics. MM helped in the experiments. PG did the assays. GA participated in the analysis of the results. IEM conceived the idea and supervised the experiments. All authors read and approved the final draft.
